# Unusual migration of implantable cardioverter defibrillator that clinically mimicking breast cancer: A case report^☆^

**DOI:** 10.1016/j.radcr.2023.11.018

**Published:** 2023-11-25

**Authors:** Abdulwahid M. Salih, Lana RA. Pshtiwan, Halkawt O. Ali, Shko H. Hassan, Vanya I. Jwamer, Shaban Latif, Jihad Ibrahim Hama, Fahmi H. kakamad

**Affiliations:** a Smart Health Tower, Sulaimani, Kurdistan, Iraq; b College of Medicine, University of Sulaymaniyah, Sulaymaniyah, Iraq; c Kscien Organization, Sulaimani, Kurdistan, Iraq; d Research Center, University of Halabja, Halabja, Kurdistan, Iraq

**Keywords:** Pacemaker, ICD, Breast cancer, Migration, Cardiac arrhythmias

## Abstract

Pacemaker and implantable cardioverter defibrillator migration to the breast are an extremely rare complication. The rarity of this phenomenon and its potential to mimic breast cancer emphasize the importance of reporting such cases. This study presents a rare migration of the device to the breast tissue that clinically mimicked breast cancer. This case underscores the need for comprehensive diagnostic approaches and individualized management strategies when faced with such clinical challenges. A 59-year-old female patient complained bilateral breast masses for a 3-month duration. She is a known case of diabetes mellitus and hypertension. In 2015, she underwent Implantable cardioverter defibrillator implantation for dilated cardiomyopathy and left ventricular failure. On examination, there was a skin dimpling in the left upper quadrant of her breast. The skin dimpling was clinically suspected to be breast cancer. Mammography showed an implantable cardiac device in the upper central part extending into the glandular parenchyma. A consultation with a cardiologist confirmed that the ICD was functioning properly, and as a result, no medical interventions were deemed required. Implantable cardioverter defibrillator migration to the breast is an extremely rare phenomenon and represent a complex clinical challenge that require a comprehensive diagnostic approach and individualized management strategies**.**

## Background

The pacemaker and implantable cardioverter defibrillators (ICD) are electronic devices that have become commonly implanted in recent years, saving millions of lives and increasing people's quality of life [1], [2]–3]. The device indications are increasing, individuals with heart disorders such as atrial fibrillation or sick sinus syndrome and arrhythmias require a pacemaker or ICD for control [4,5]. As with any other surgical procedure or foreign body implantation, complications have been reported in 3%-7% of ICD patients, including migration, dislocation, explosion once heated, and in a few documented instances, the development of cancer within the ICD pocket [6,7]. The left ventricle is the most common site of ICD migration through the interventricular septum, the device can migrate to any location, such as the abdominal cavity or the lungs. ICD and pacemaker migration to the breast are extremely rare complications. This phenomenon involves the displacement of the pacemaker generator, leads, or both from their intended location within the chest to the breast tissue [8]. The rarity of this phenomenon and its potential to mimic breast cancer emphasizes the importance of reporting such cases. Understanding these occurrences can contribute to improved patient care and advance current knowledge of this uncommon complication. This condition underscores the need for comprehensive diagnostic approaches and individualized management strategies when faced with such clinical challenges. This case report delves into a unique and clinically significant event—ICD migration to the breast.

## Case presentation

*Patient information:* A 59-year-old non-smoking female patient presented to the clinic complaining of bilateral breast masses and mastalgia for 3 months. She has seven children with 3 years of lactation. She is a known case of diabetes mellitus (DM), hypertension (HTN), heart disease, and psychiatric disorders. She had a family history of breast cancer (her nephew) and gastric cancer (her mother). She had previously undergone kidney surgery in 1997, a right breast lumpectomy in 2018 for fibroadenoma, and ICD implantation surgery twice for dilated cardiomyopathy and left ventricular failure, once in 2015, followed by another after 5 months due to infection. She routinely visited her cardiologist on schedule and had no trouble with her pacemaker.

**Fig. 1:**
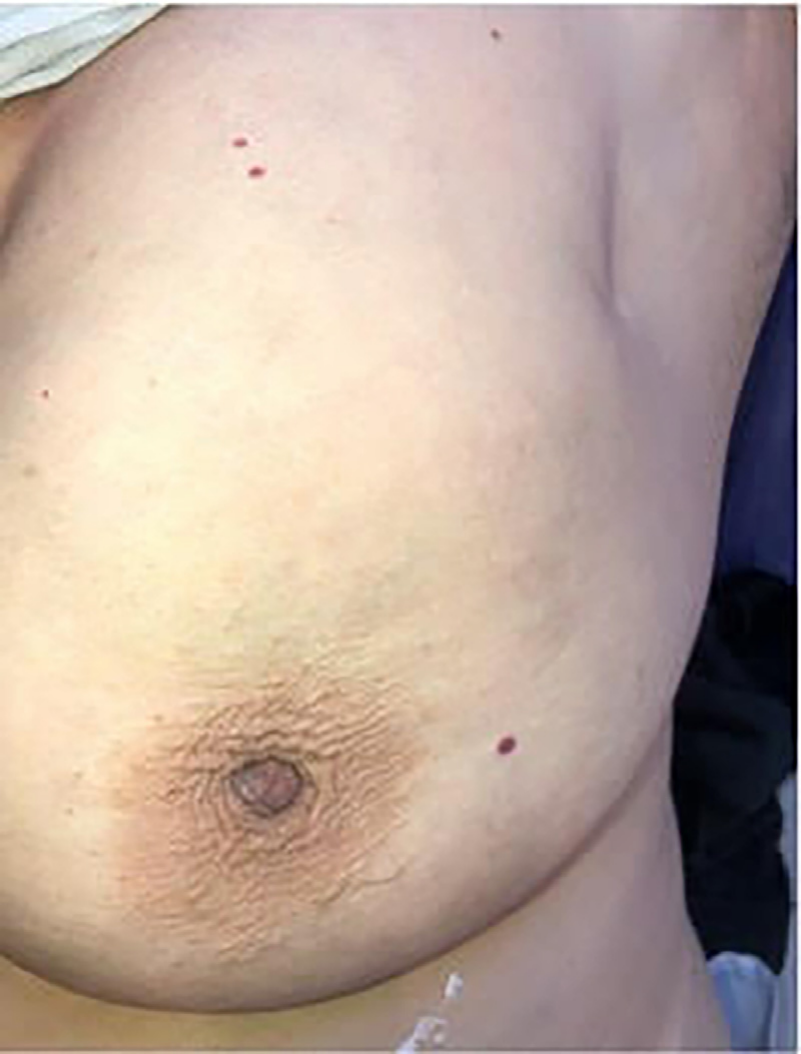
Skin dimpling in the left upper outer quadrant of the breast.

*Clinical examination:* On examination, the breasts were symmetrical, with no variation in shape or size. Both breasts looked normal in color. There was no visible lesion or ulceration, the nipple and areola seemed normal, and there was no spontaneous discharge or discharge on squeezing, only skin dimpling in the left upper outer quadrant (Fig. 1). On palpation, there was no temperature change, tenderness, or visible or palpable tumor. The skin dimpling was clinically suspected to be cancer.

**Fig. 2:**
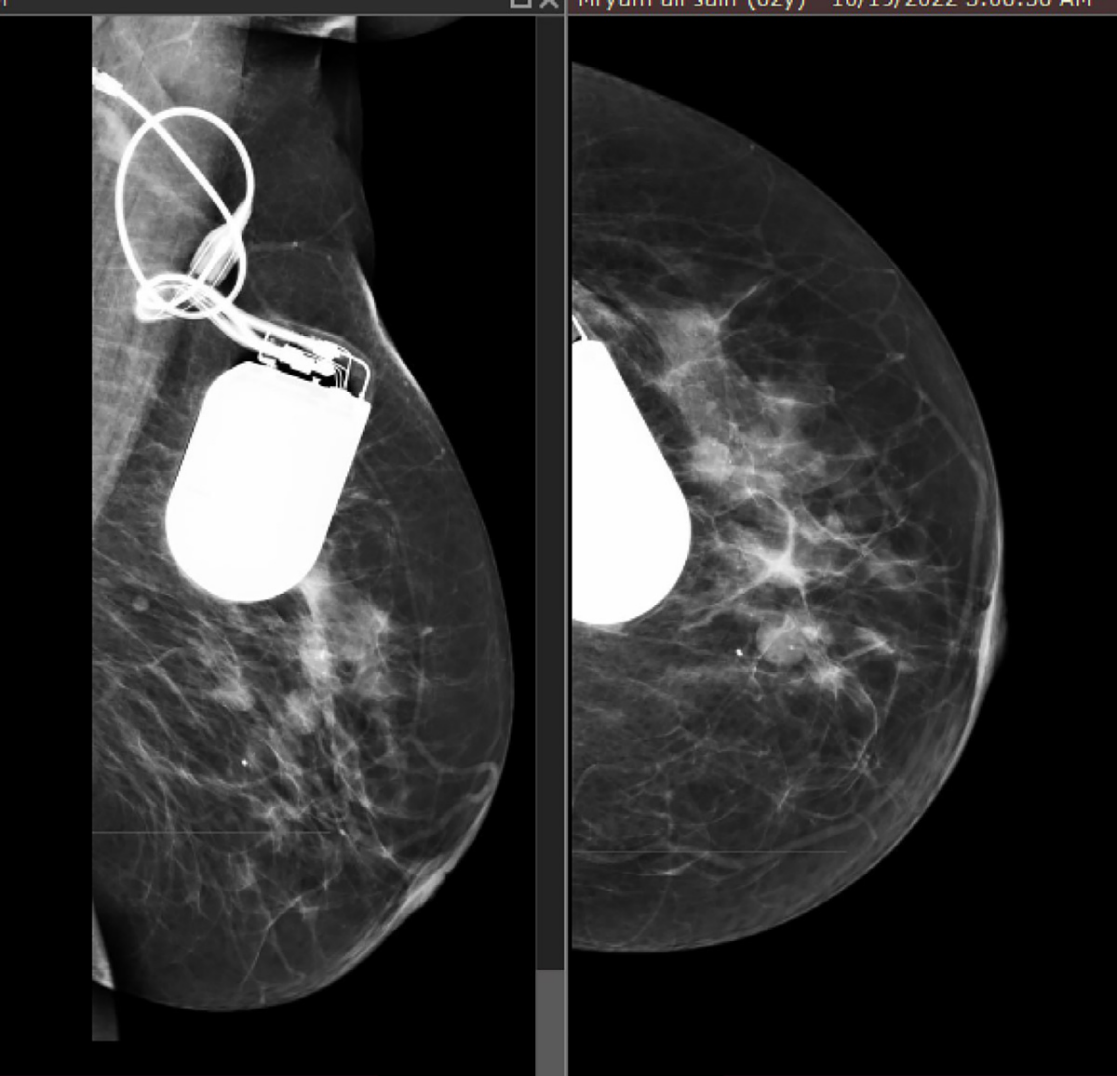
Mammogram -MLO and CC View of the left breast, showing cardiac ICD in the upper central part extending into the glandular parenchyma.

**Fig. 3:**
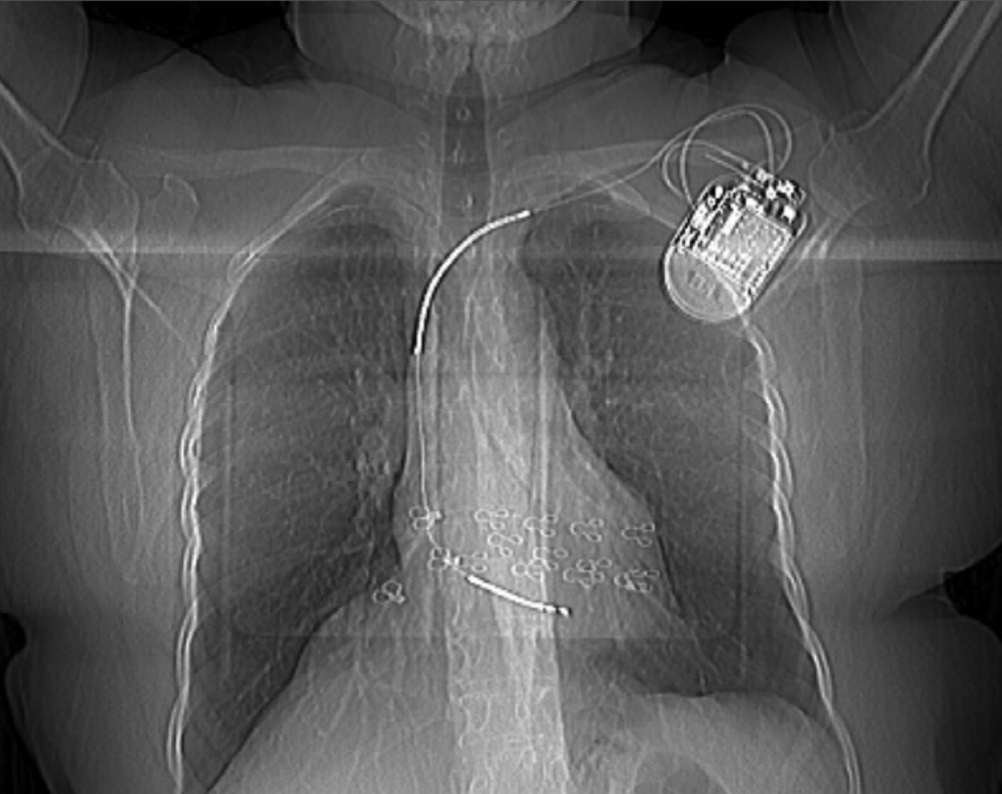
Scout image of Chest CT scan showing a single chamber ICD implant in the left upper chest wall.

**Fig. 4:**
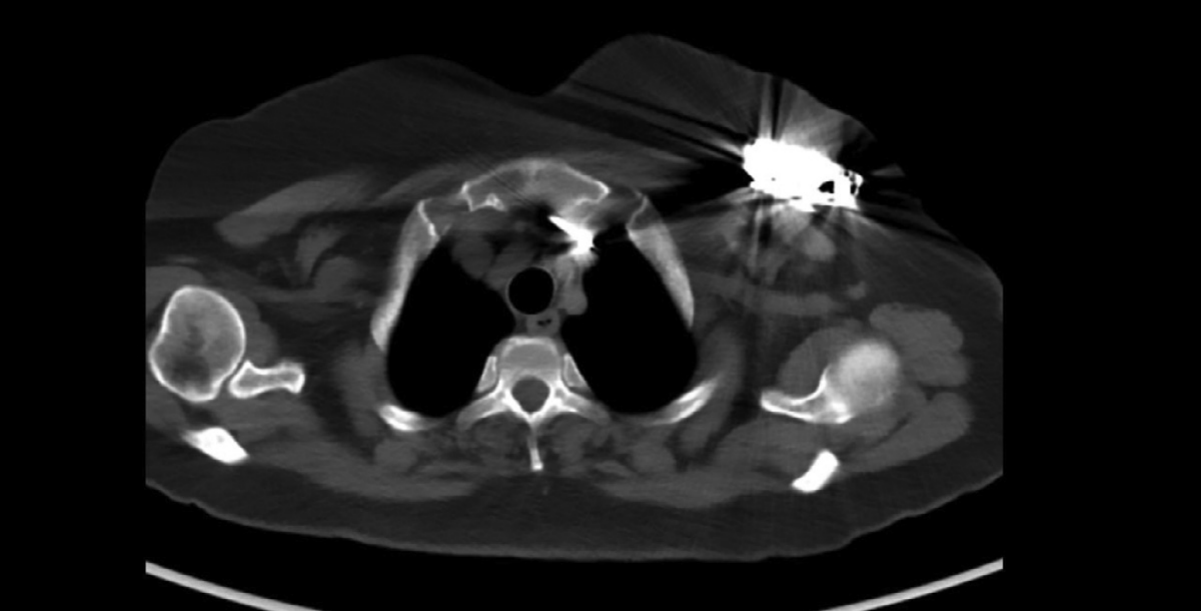
CT scans axial view show metal artifact in the left infra-clavicular region corresponding to a cardiac ICD.

*Diagnostic intervention****:*** On ultrasound, there were multiple simple cysts in both breasts measuring 5-10 mm with spots of calcification, with no suspicious mass or distortion, normal nipples, and normal skin thickness. There was a cardiac device in the upper central portion of the left breast, located 2 cm deep into the skin. Mammography showed a cardiac device in the upper central part extending into the glandular parenchyma (Fig. 2). A scout image of a chest computed tomography (CT) scan showed a single chamber cardiac device implant in the left upper chest wall (Fig. 3). A CT scan axial view showed a metal artifact in the left infra-clavicular region corresponding to the cardiac device (Fig. 4).

*Therapeutic intervention:* A cardiologist was consulted regarding the case, and it was determined that there were no issues with the ICD or its functioning. No therapeutic interventions were deemed necessary. The patient's consent was obtained for ongoing follow-up, and she continued to receive regular breast examinations as part of her routine healthcare. If the device were to exhibit any issues in the future, the option of surgical repositioning of the device remains available.

*Follow-up:* Over the course of the 6-month follow-up, there was no evidence of new migration of the device, and the patient tolerated the presence of the device well. The patient has not reported any new complaints or complications during the follow-up period. Furthermore, the cardiac device has continued to function effectively without any signs of malfunction. This indicates a favorable outcome for the patient in terms of both device performance and overall well-being.

## Discussion

ICD and pacemakers have revolutionized the management of cardiac arrhythmias, improved patients' quality of life, and reduced morbidity and mortality rates. Complications such as migration, dislodgment, and sometimes perforation may occur [9]. ICD migration can occur due to various factors. One primary cause is inadequate fixation or displacement of the ICD during the initial implantation surgery. Factors contributing to inadequate fixation include poor surgical technique, excessive subcutaneous tissue, and insufficient anchoring of the device. Additionally, inadvertent dislodgment may result from traumatic incidents, such as falls or accidents, that cause mechanical disruption of the device pocket [10,11]. The current patient has had 2 previous operations, which increased the likelihood of ICD migration.

Although ICD migration to the left ventricle through the septum is the most common, there have been reports of migration to the Douglas pouch, hepatic vasculature, or urinary bladder, which can result in partial or full intestinal blockage, colonic perforation, or death [12], [13], [14], [15], [16]–17]. ICD migration to breast tissue is a seldom-documented phenomenon in the medical literature. However, a case of subcutaneous ICD migration to the breast has been reported. This migration occurred due to the positioning of the device and implant under the pectoral muscle, coupled with the absence of secure device fixation [18]. In the current case, ICD migrated to the breast.

The presentation of this migration differs depending on the place of migration. The most common sign is an empty pocket, indicating that the ICD cannot be found at the insertion site [18]. Diagnosing ICD migration to the breast can be challenging due to the relatively low incidence of this phenomenon and the potential for nonspecific symptoms. Patients may present with pain, discomfort, a palpable mass, or visible displacement of the device. However, these symptoms can be mistaken for other benign or malignant breast conditions, necessitating a comprehensive diagnostic workup. In the present case, there was a skin dimpling, which was initially considered to be breast cancer.

Commonly employed suspicious findings or breast mass diagnostic modalities include physical examination and imaging techniques (mammography, ultrasound, or magnetic resonance imaging) [19], [20]–21]. In the current case, ultrasound showed a pacemaker in the upper central portion of the left breast, located 2 cm deep into the skin. Mammography showed a cardiac device in the upper central part extending into the glandular parenchyma.

Device migration to the breast possesses several clinical implications. Firstly, there is a risk of interference with the device's function, leading to inadequate pacing or complete failure. This can result in recurrent arrhythmias, syncope, or hemodynamic compromise [20]. Secondly, as in the current case, physical displacement of the device might cause the patient discomfort, pain, and emotional distress. Furthermore, the existence of a visible or palpable breast lump or other findings raises the possibility of cancer. Additionally, it is important to acknowledge the potential psychological impact on the patient. The discovery of an unusual mass in the breast and the need for diagnostic procedures can cause anxiety, stress, and emotional distress. Understanding and addressing these psychological aspects are crucial for holistic patient care.

The management of device migration to the breast is multifaceted and requires a multidisciplinary approach. The first step is establishing an accurate diagnosis through clinical assessment, imaging, and device interrogation. Once confirmed, the management strategy depends on the clinical presentation and individual patient factors [22,23]. Conservative management, involving close monitoring and surveillance, may be appropriate for asymptomatic patients with a well-functioning device. However, symptomatic patients, compromised device function, or suspicion of malignancy may necessitate intervention, such as surgery for repositioning of device because it's the first line or the use of a matrix for soft tissue stabilization [18–20]. Since the ICD was functioning well without any significant symptoms, the patient provided consent, and, following reassurance, the current case only underwent routine breast follow-up examinations.

This case highlights the psychological distress caused by ICD migration to the breast, which can lead patients to fear breast cancer. It underscores the need for a thorough diagnostic approach to distinguish benign conditions from breast malignancies. ICD migration can disrupt device function, potentially resulting in arrhythmias, syncope, or discomfort for the patient. To prevent migration, surgeons should use precise implantation techniques. Patient education is vital for early issue recognition. Regular check-ups are essential for device monitoring, and comprehensive diagnostic evaluations are necessary when breast-related symptoms arise.

## Conclusion

ICD migration to the breast is an extremely rare phenomenon. It represents a complex clinical challenge that requires a comprehensive diagnostic approach and individualized management strategies, considering the patient's symptoms, device function, and the presence of suspicious breast findings. The condition can lead to patient discomfort and potential diagnostic confusion with breast malignancies. Prompt recognition is crucial for optimized patient outcomes. Collaborative efforts among cardiologists and surgeons are essential for successfully managing this rare but important clinical entity.

## Consent for publication

Not applicable.

## Availability of data and material

All data and materials are kept by the first and corresponding authors.

## Ethics approval

Not applicable

## Authors' contributions

Abdulwahid M. Salih: major contribution of the idea, and the surgeon performed operation final approval of the manuscript. Halkawt O. Ali, Jihad Ibrahim Hama and Shaban Latif: were a major contributor to the conception of the study, as well as in the literature search for related studies Lana RA. Pshtiwan: The radiologist who performing assessment of the case. Shko H. Hassan and Fahmi H. kakamad: confirm the authenticity of all the raw data and final approval of the manuscript. Fahmi H. kakamad and Vanya I. Jwamer: Writing the manuscript, literature review, final approval of the manuscript. All authors have read and approved the final manuscript.

## Patient consent

Consent has been taken from the patients and the family of the patients.
